# Mapping the neurovascular landscape in aging and dementia: cerebral small vessel disease markers in a multicenter Latin American cohort

**DOI:** 10.1002/alz.71468

**Published:** 2026-05-26

**Authors:** Florencia Altschuler, Ana Maria Castro‐Laguardia, Verónica Canziani, Daniel Franco O'Byrne, Jessica Hazelton, Joaquín Migeot, Marcelo Maito, Adolfo M. García, Martín A. Bruno, Nahuel Magrath, Andrea Slachevsky, María Isabel Behrens, Nilton Custodio, José Alberto Ávila‐Funes, Diana Matallana, David Aguillón, Hernando Santamaria‐García, Elisa Resende, Leonel Takada, Luis Ignacio Brusco, Jennifer S. Yokoyama, Bruce Miller, Agustín Ibáñez, Vicente Medel, Cecilia Gonzalez Campo

**Affiliations:** ^1^ Cognitive Neuroscience Center Universidad de San Andrés Victoria Buenos Aires Argentina; ^2^ Consejo Nacional de Investigaciones Científicas y Técnicas (CONICET) Ciudad Autónoma de Buenos Aires Argentina; ^3^ Facultad de Medicina Universidad de Buenos Aires Ciudad Autónoma de Buenos Aires Argentina; ^4^ Latin American Brain Health Institute (BrainLat) Universidad Adolfo Ibañez Santiago de Chile Región Metropolitana Chile; ^5^ Cognitive and Affective Neuroscience Center. Universidad Adolfo Ibáñez Santiago de Chile Región Metropolitana Chile; ^6^ Brain and Mind Centre The University of Sydney Camperdown, Sydney Australia; ^7^ School of Health Sciences The University of Sydney Camperdown, Sydney Australia; ^8^ Brain Health Institute (GBHI) Trinity College Dublin Dublin Ireland; ^9^ Global Brain Health Institute (GBHI) University of California San Francisco California USA; ^10^ Departamento de Lingüística y Literatura Facultad de Humanidades Universidad de Santiago de Chile Santiago Región Metropolitana Chile; ^11^ Instituto de Ciencias Biomédicas Universidad Católica de Cuyo San Juan Argentina; ^12^ Geroscience Center for Brain Health and Metabolism Santiago de Chile Región Metropolitana Chile; ^13^ Memory and Neuropsychiatric Center (CMYN) Neurology Department, Hospital del Salvador & Faculty of Medicine Universidad de Chile Santiago Región Metropolitana Chile; ^14^ Neuropsychology and Clinical Neuroscience Laboratory (LANNEC), Facultad de Medicine Universidad de Chile Santiago Región Metropolitana Chile; ^15^ Departamento de Neurología y Psiquiatría Clínica Alemana, Universidad del Desarrollo Santiago Región Metropolitana Chile; ^16^ Centro de Investigación Clínica Avanzada (CICA) and Departamento de Neurología y Neurocirugía Departamento de Neurociencia Facultad de Medicina, Hospital Clínico Universidad de Chile Santiago Región Metropolitana Chile; ^17^ Unit Cognitive Impairment and Dementia Prevention, Peruvian Institute of Neurosciences Lima Peru; ^18^ Geriatrics Department Instituto Nacional de Ciencias Médicas y Nutrición Salvador Zubirán Ciudad de México México; ^19^ Centro de Memoria y Cognición Intellectus Hospital Universitario San Ignacio Bogotá Colombia; ^20^ PhD Program of Neuroscience, Psychiatry Department Pontificia Universidad Javeriana Bogotá Colombia; ^21^ Mental Health Department Hospital Universitario Fundación Santa Fe Bogotá Colombia; ^22^ Grupo de Neurociencias de Antioquia Universidad de Antioquia Medellín Colombia; ^23^ Universidade Federal de Minas Gerais, Hospital das Clínicas ‐ EBSERH‐ UFMG Belo Horizonte Minas Gerais Brazil; ^24^ Grupo de Neurologia Cognitiva e do Comportamento (GNCC) Hospital das Clinicas Faculdade de Medicina da Universidade de São Paulo São Paulo Brazil; ^25^ Departamento de Psiquiatría y Salud Mental, Facultad de Medicina Universidad de Buenos Aires Ciudad Autónoma de Buenos Aires Argentina; ^26^ Edward and Pearl Fein Memory and Aging Center Department of Neurology Weill Institute for Neurosciences University of California San Francisco California USA; ^27^ Department of Radiology and Biomedical Imaging University of California San Francisco California USA; ^28^ Department of Biophysics School of Medicine Istanbul Medipol University Istanbul Türkiye; ^29^ Barcelonaβeta Brain Research Center (BBRC) Pasqual Maragall Foundation Barcelona Spain; ^30^ Facultad de Ciencias Biológicas Pontificia Universidad Católica de Chile Santiago Región Metropolitana Chile

**Keywords:** Alzheimer's disease, cardiometabolic risk factors, cerebral small vessel disease, dementia, frontotemporal dementia, Latin America

## Abstract

**INTRODUCTION:**

Cerebral small vessel disease (CSVD) is a key contributor to cognitive impairment and dementia, yet few studies have compared CSVD across dementia variants, particularly in underrepresented populations.

**METHODS:**

In a multicenter cross‐sectional study, we analyzed magnetic resonance imaging (MRI) markers of CSVD, including white matter hyperintensities (WMHs), lacunes, and cerebral microbleeds, along with cardiometabolic risk factors and cognitive performance using regression models in 1675 participants (790 healthy controls, 642 with Alzheimer's disease [AD], and 243 with frontotemporal dementia [FTD]) from six Latin American countries.

**RESULTS:**

AD showed the greatest CSVD burden, whereas FTD exhibited an intermediate profile driven by elevated WMHs. Blood pressure and smoking were the strongest correlates of WMHs, while diabetes was associated with microbleeds. WMH burden was linked to global and domain‐specific cognitive impairment.

**DISCUSSION:**

This first large‐cohort Latin American study identifies WMHs as a key vascular substrate of cognitive impairment, with AD showing the greatest CSVD burden.

## Highlights

- This is the first large‐scale characterization of CSVD across aging, AD, and FTD in Latin America.
- WMHs are the most prevalent CSVD marker and the strongest predictor of cognition.
- AD shows the highest CSVD burden, while FTD presents an intermediate vascular profile.
- BP is the main vascular correlate of WMHs across diagnostic groups.
- Findings support heterogeneous, context‐sensitive models of CSVD in dementia.

## 1 BACKGROUND

Cerebral small vessel disease (CSVD) is a common disorder affecting the brain's microvasculature and represents a major contributor to stroke,^1^, ^2^ cognitive decline, and dementia worldwide.^1^, ^3^, ^4^, ^5^, ^6^ CSVD is closely linked to cardiovascular risk (CVR) factors, particularly hypertension,^1^ but also other lifestyle factors^5^, ^7^ related to endothelial dysfunction and atherosclerosis.^2^, ^7^, ^8^ On magnetic resonance imaging (MRI), CSVD is primarily characterized by white matter hyperintensities (WMHs), lacunes, and microbleeds, reflecting different but interrelated mechanisms of microvascular injury.^6^, ^9^ WMHs correspond to areas of demyelination and axonal loss related to chronic hypoperfusion and blood–brain barrier dysfunction, while lacunes represent small cavitated infarcts caused by occlusion of deep perforating arteries.^10^ Cerebral microbleeds are microhemorrhages resulting from arteriolar fragility, most commonly associated with hypertensive vasculopathy or cerebral amyloid angiopathy.^11^

CSVD is highly prevalent at the population level, with MRI evidence frequently observed even in cognitively normal middle‐aged and older adults.^4^ Although CSVD does not invariably lead to cognitive impairment, longitudinal studies show that greater CSVD burden is associated with accelerated cognitive decline and increased risk of dementia.^3^, ^12^ The cognitive profile of CSVD involves deficits in executive function, processing speed, and global cognition, reflecting disruption of frontal‐subcortical networks.^13^ Alongside AD, CSVD is recognized as one of the most common contributors to dementia worldwide and frequently coexists with neurodegenerative pathology, amplifying cognitive vulnerability.^3^ Neuropathological studies from Europe and North America show that mixed vascular and neurodegenerative pathology is present in 30% to 50% of dementia cases in community‐based autopsy cohorts, with vascular lesions contributing additively to cognitive impairment.^14^, ^15^ These lesions are particularly associated with executive dysfunction and processing speed deficits,^16^ and vascular cognitive impairment accounts for approximately 20% to 40% of dementia diagnoses globally.^17^ Similar findings have been reported in Latin America, where cerebrovascular pathology frequently co‐occurs with Alzheimer‐type changes in dementia cases.^18^, ^19^, ^20^

In this context, understanding the vascular mechanisms underlying CSVD is essential, as CVR factors play a central role in its development and progression. Hypertension, including systolic and diastolic blood pressure (BP) abnormalities, consistently emerges as the strongest determinant of WMHs, lacunes, and deep cerebral microbleeds.^7^, ^21^ Other factors such as smoking, diabetes, obesity, dyslipidemia, and systemic atherosclerosis are also associated with CSVD burden, though with greater variability across populations.^22^ Associations are lesion‐specific: deep lacunes and microbleeds are primarily linked to hypertensive arteriopathy, whereas lobar microbleeds more often reflect cerebral amyloid angiopathy.^5^, ^11^, ^23^

Most CSVD research has been conducted in cohorts from Europe and North America, with limited representation from other regions, including low‐ and middle‐income countries.^24^, ^25^ This geographic imbalance constrains generalizability across diverse environmental and vascular contexts. Moreover, investigations have predominantly focused on AD, where microvascular pathology exacerbates neurodegenerative vulnerability.^26^ In contrast, the role of CSVD in non‐AD dementias is less characterized. Evidence suggests CSVD may influence disease severity and functional outcomes in frontotemporal dementia (FTD)^27^, ^28^ and contribute to cognitive impairment in early‐onset dementia syndromes.^29^ Nevertheless, targeted investigations of CSVD in FTD and other non‐AD dementias remain scarce, representing a critical gap in the literature.

These gaps are particularly relevant in Latin America, a region undergoing rapid population aging alongside a high prevalence of vascular risk factors, including hypertension, obesity, diabetes, and smoking.^30^ Despite this convergence of risk, Latin American populations remain underrepresented in CSVD research, and it remains unclear whether associations observed in high‐income countries generalize to this region. To address these gaps, this study characterizes CSVD in a large Latin American cohort by quantifying WMH, lacunes, and microbleeds in cognitively healthy older adults, individuals with AD, and those with FTD. We further examine associations between CSVD burden, CVR factors, and cognitive performance, aiming to advance understanding of microvascular contributions to cognitive aging and dementia in an understudied population.

## 2 METHODS

**FIGURE 1 alz71468-fig-0001:**
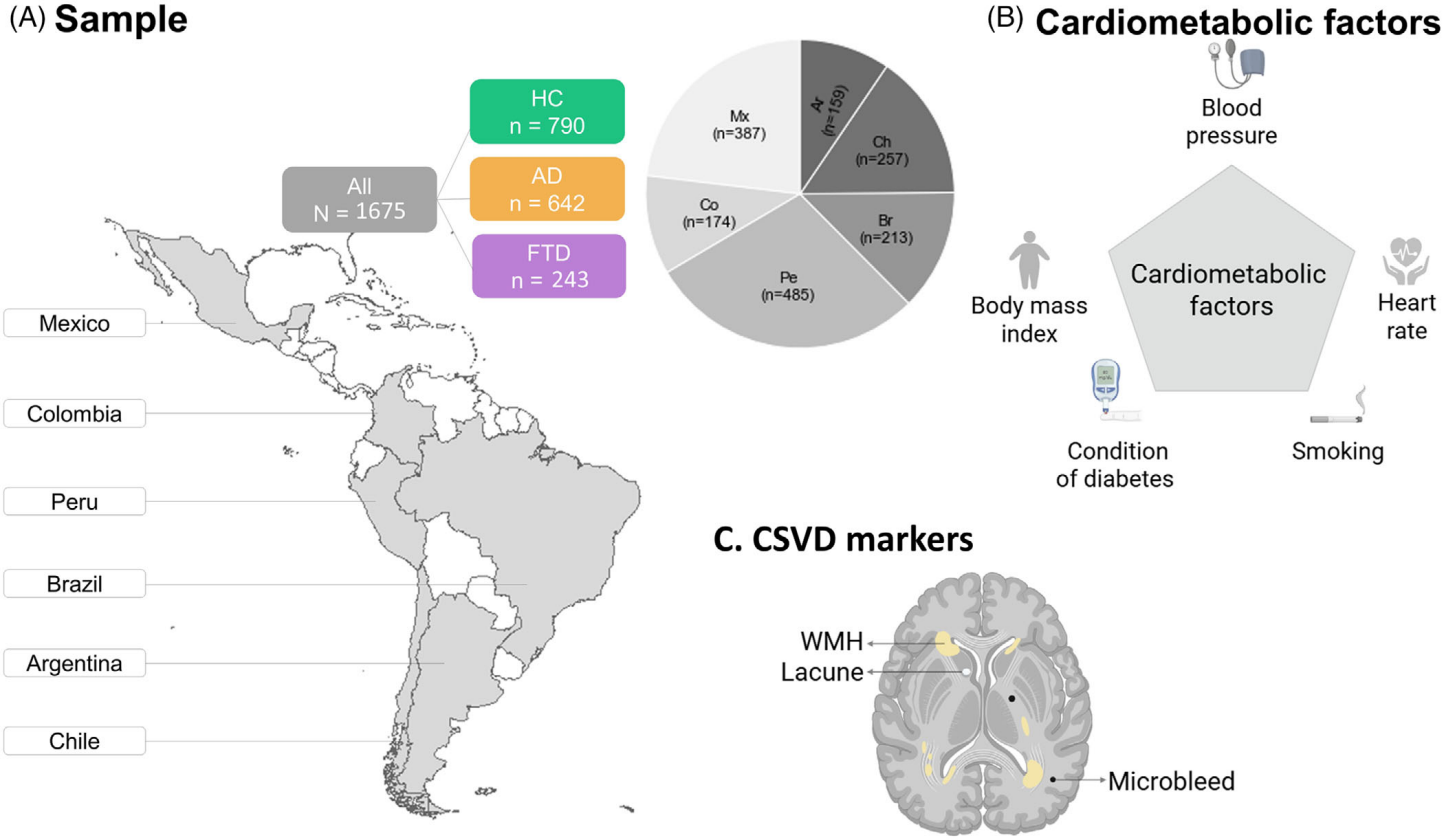
Study workflow and sample composition. (A) Sample characterization, including diagnostic groups and country‐level distribution (pie charts indicate proportional representation from each participating country). (B) Cardiometabolic factor and (C) CSVD markers. AD, Alzheimer's disease; CSVD, cerebral small vessel disease; FTD, frontotemporal dementia; HC, healthy controls; Mx, Mexico; Co, Colombia; Pe, Peru; Br, Brasil; Ar, Argentina; Ch, Chile.

This study employed a multicenter, cross‐sectional design to investigate structural neuroimaging markers and their associations with cardiometabolic variables and cognitive variables across healthy aging and dementia subjects from six Latin American countries. The study's methods are summarized in Figure 1.

### 2.1 Participants

The study comprised 1675 participants (see Table 1 for demographic details), including 790 healthy controls (HCs), 642 individuals with AD, and 243 with FTD. Participants were recruited through the Multi‐Partner Consortium to Expand Dementia Research in Latin America (ReDLat),^31^ which integrates data from nine sites across six countries (Argentina, Brazil, Chile, Colombia, Mexico, and Peru). All participants were between 40 and 80 years of age, demonstrated sufficient language fluency, and had adequate visual and auditory abilities to complete neuropsychological testing. HCs exhibited preserved cognition (Clinical Dementia Rating = 0; Mini‐Mental State Examination [MMSE] > 25) and no history of major cognitive, neurological, or psychiatric illness or substance abuse.

**TABLE 1 alz71468-tbl-0001:** Demographic and cognitive characteristics of sample.

Variable	All (*n* = 1675)	HC (*n* = 790)	AD (*n* = 642)	FTD (*n* = 243)
**Age**	66.2 (9.8)	62.2 (9.7)	71.3 (7.7)	66.1 (8.3)
**Sex**	526 (32.7%) M, 1088 (67.3%) F	199 (26.2%) M, 570 (73.8%) F	209 (33.8%) M, 408 (66.2%) F	118 (50.6%) M, 110 (49.4%) F
**Years of education**	12.1 (5.6) *n* = 1604	12.4 (5.9) *n* = 762	11.7 (5.3) *n* = 615	12.7 (5.0) *n* = 227
**MMSE**	23.9 (5.3) *n* = 1612	27.2 (3.2) *n* = 769	20.8 (4.8) *n* = 616	21.4 (6.2) *n* = 227

*Note*: Values are presented as mean (standard deviation) for continuous variables and as number (percentage) for categorical variables. Sample sizes are indicated for variables with missing data.

Abbreviations: AD, Alzheimer's disease; F, female; FTD, frontotemporal dementia; HC, healthy controls; M, male; MMSE, Mini‐Mental State Examination.

AD diagnosis followed the current clinical diagnostic frameworks, including the typical amnestic presentation^32^ and atypical variants characterized by predominant language,^33^ visuospatial,^34^ or behavioral features.^35^ FTD diagnoses were established according to contemporary clinical criteria encompassing behavioral, personality, and/or language disturbances,^33^, ^36^ as well as motor presentations.^37^, ^38^, ^39^

**RESEARCH IN CONTEXT**

1. **Systematic review**: CSVD is a major contributor to cognitive impairment and dementia. However, Latin American populations remain markedly underrepresented in CSVD research, despite a high prevalence of cardiometabolic risk factors and rapid population aging. Moreover, most prior work focused on AD, with limited investigation of CSVD in non‐AD dementias such as FTD.
2. **Interpretation**: In a large multicenter Latin American cohort, CSVD markers were common across healthy older adults and dementia syndromes, but their distribution and clinical relevance varied by diagnosis. WMH represented the most robust and integrative marker, strongly associated with BP and cognitive impairment across groups. AD exhibited the highest overall CSVD burden, while FTD showed a distinct intermediate profile, underscoring that CSVD phenotypes are not interchangeable across dementias. These results suggest that CSVD reflects multiple, partially dissociable vascular processes shaped by diagnostic vulnerability and population context.
3. **Future directions**: Longitudinal studies are needed to determine how CSVD markers contribute to disease progression and cognitive decline over time in Latin American populations. Future research should integrate social, environmental, and life‐course exposures to better capture how contextual factors shape neurovascular aging. Expanding CSVD research beyond AD and into underrepresented regions will be critical for developing more accurate, population‐sensitive models of vascular contributions to dementia and for informing targeted prevention strategies.

Exclusion criteria applied to all participants included the presence of brain tumors or large, clinically significant ischemic or hemorrhagic cerebral infarcts on MRI, as well as significant systemic medical conditions (including vitamin B12 deficiency, hypothyroidism, human immunodeficiency virus infection, renal, hepatic, or respiratory failure requiring oxygen) and inability to read and write.

The study was approved by the Institutional Review Boards of each recruitment site and the Executive Committee of the ReDLat consortium. All participants or their caregivers provided informed consent in line with the Declaration of Helsinki. Global cognitive functioning was assessed using the MMSE, the widely used screening tool for detecting cognitive impairment.[Fig alz71468-fig-0001], [Table alz71468-tbl-0001]

### 2.2 Cardiometabolic assessment

Cardiometabolic variables were systematically recorded for all participants as part of the clinical assessment. These measures included systolic and diastolic BP, heart rate, diabetes status, body mass index (BMI), and cumulative tobacco exposure expressed as pack‐years of smoking. BP and heart rate were obtained under standardized resting conditions, while diabetes status was determined based on clinical history and/or medical records. BMI was calculated from height and weight measurements, and the smoking exposure (pack‐years) was quantified by combining duration of tobacco use and number of cigarettes consumed per day. These variables were included to characterize the cardiometabolic profile of the sample and to account for vascular and metabolic factors known to influence brain structure and cognitive outcomes.

### 2.3 Neuroimaging acquisition, preprocessing, and analyses

Whole‐brain 3D T1‐weighted and Fluid‐Attenuated Inversion Recovery (FLAIR) sequences were acquired for all participants across acquisition centers. Susceptibility‐weighted imaging (SWI) sequences were acquired for a subset of participants (*n* = 475). Participants with cavernomas, brain tumors, or scans with excessive noise or artifacts were excluded from the analyses. Detailed scanning protocols for each center are provided in Table S1.

Structural T1‐weighted images were preprocessed with the Computational Anatomy Toolbox (CAT12; www.neuro.uni-jena.de/cat/) implemented in Statistical Parametric Mapping software (SPM 12; Wellcome Centre for Human Neuroimaging; www.fil.ion.ucl.ac.uk/spm/software/spm12/)^40^ on MATLAB R2017b. Preprocessing steps included skull stripping, gray matter (GM) and white matter (WM) segmentation, and normalization to a 1.5‐mm structural Montreal Neurological Institute (MNI) template.^41^ Total intracranial volume (TIV) was calculated by summing the raw volumes of GM, WM, and cerebrospinal fluid.

WMH segmentation was performed using the lesion prediction algorithm (LPA; http://www.applied-statistics.de/lst.html),^42^ implemented in the LST toolbox, version 2.0.15, for SPM. Individual FLAIR images were used to obtain lesion probability maps, which were visually inspected for artifacts and discarded if artifacts were present (commonly found in the choroid plexus and basal cisterns). Total WMH volume (in milliliters) was extracted from subject‐level WMH probability maps using the default threshold of 0.5 and normalized by TIV.

Lacunes and cerebral microbleeds were visually assessed by one neuroradiologist (CGC) and two trained researchers (FA, VC) according to the STandards for Reporting Vascular changes on nEuroimaging (STRIVE) criteria.^9^, ^10^ Lacunes were defined as focal lesions measuring 3 to 15 mm in diameter, isointense to cerebrospinal fluid on T1‐weighted and FLAIR images, typically with a peripheral hyperintense rim on FLAIR. Lesion morphology and location were used to differentiate lacunes from perivascular spaces, with lacunes typically showing a round or ovoid shape. In contrast, perivascular spaces generally follow a linear shape along perforating arteries.

Cerebral microbleeds were identified in SWI images, defined as small, low‐signal, circular lesions measuring 2 to 5 mm in diameter. Microbleeds were classified by location as deep (basal ganglia, thalamus, corpus callosum, brain stem, and central cerebellum), peripheral (frontal, parietal, temporal, occipital, and cerebellar), or both.

All analyses were conducted blind to clinical diagnosis.

### 2.4 Statistical analysis

Demographic, cognitive, cardiometabolic, and imaging variables were compared across diagnostic groups (HC, AD, FTD) using a Kruskal–Wallis test for continuous variables and chi‐squared or Fisher's exact tests for categorical variables when expected cell counts were <5. When global tests were significant, pairwise post hoc comparisons were performed using Dunn's tests with Holm correction for multiple comparisons. All tests were two‐tailed, and statistical significance was set at *p* < 0.05.

To evaluate demographic associations with CSVD markers, regression models were fitted with age and sex as predictors for each CSVD outcome. In the case of WMH volume, linear regression models were employed, whereas the presence of lacunes and microbleeds was analyzed using logistic regression models. WMH models were additionally adjusted for TIV. These demographic models were fitted in the full sample and within each diagnostic group.

Associations among CSVD markers were examined in the full sample and stratified by diagnostic group. Differences in WMH burden according to the presence or absence of lacunes or microbleeds were assessed using Mann–Whitney U tests. Co‐occurrence of lacunes and microbleeds were tested using Pearson's chi‐squared tests.

Associations between vascular risk factors and CSVD markers were examined using regression models. Vascular predictors included systolic and diastolic BP, heart rate, diabetes, BMI, and smoking exposure (pack‐years). Continuous vascular variables were standardized before analysis. Binary CSVD outcomes (presence or absence of lacunes and cerebral microbleeds) were modeled using logistic regression, while WMHs were analyzed using linear regression. All models were adjusted for age and sex; analyses in the full sample were additionally adjusted for clinical diagnosis, and WMH models were further adjusted for TIV. Separate multivariable models were fitted for each CSVD outcome (WMH, lacunes, microbleeds), including all cardiometabolic risk factors as predictors, in the full sample and within each diagnostic group (HC, AD, FTD). Effect estimates are reported as odds ratios (ORs) for binary outcomes and standardized regression coefficients (*β*) for WMHs, with 95% confidence intervals.

Associations between CSVD markers and cognitive performance were examined using linear regression models with global cognition (MMSE) and domain‐specific cognitive composite scores as outcomes (see Supplementary Methods for details on composite score construction). WMH burden was expressed relative to TIV (WMH/TIV) to account for individual differences in brain size. Models were adjusted for age, sex, and years of education, and analyses in the full sample additionally included diagnostic group. Separate models were also fitted within each diagnostic group. To account for multiple comparisons, *p* values for each CSVD marker were corrected for multiple comparisons using the false discovery rate (FDR) procedure within each cognitive outcome, diagnostic group, and model adjustment. All statistical analyses were conducted using R (version 4.4.1).

## 3 RESULTS

### 3.1 Demographic and cognitive assessment of whole sample and across diagnostic groups

The mean age of the cohort was 66.2 ± 9.8 years, with significant differences between groups. Participants with AD were significantly older than both the HC and FTD groups (*p* < 0.001 for all pairwise comparisons). Women were the majority in HCs and AD, and sex distribution differed significantly across diagnostic categories (HC vs AD: *p* = 0.002; HC vs FTD: *p* < 0.001; AD vs FTD: *p* < 0.001). Overall, the sample had a mean of 12.1 ± 5.6 years of education, with AD participants showing significantly fewer years of formal education than both HC (*p* < 0.001) and FTD (*p* = 0.024). Cognitive performance, assessed with the MMSE, was markedly lower in both dementia groups relative to HC (*p* < 0.001). Post hoc analyses further revealed that AD participants had slightly lower MMSE scores than those with FTD (*p* = 0.004). For a detailed description of the demographic and cognitive variables, see Table 1.

### 3.2 CSVD markers

**FIGURE 2:**
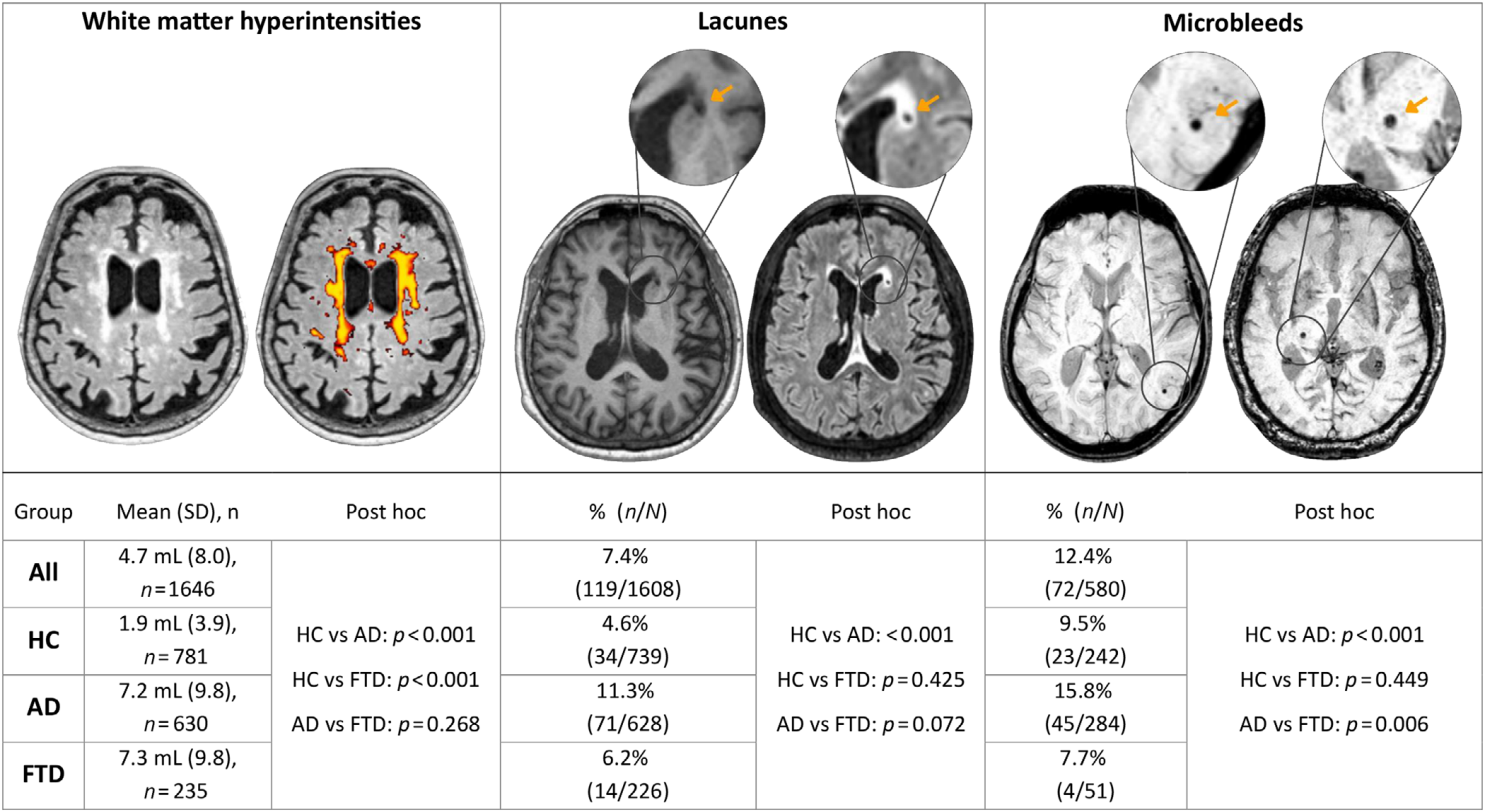
Cerebral small vessel disease markers. Top panel: examples of CSVD detection. (A) Axial FLAIR images showing a raw FLAIR image example (left) and the corresponding WMH LPA estimation mask example (right). (B) Axial FLAIR (left) and T1‐weighted (right) images showing an example of a lacune in the head of the right caudate nucleus. (C) Axial SWI showing examples of a central microbleed (left) and a peripheral microbleed (right). Bottom panel: CSVD quantification. (A) WMH burden (mL, mean ± SD), (B) percentage of participants with lacunes, and (C) percentage of participants with microbleeds. *P* values for post hoc analyses are shown for three types of comparisons: HC versus AD, HC versus FTD, and AD versus FTD. Pairwise post hoc comparisons were performed using Dunn's test with Holm adjustment for multiple comparisons. AD, Alzheimer's disease; FLAIR, Fluid‐Attenuated Inversion Recovery; FTD, frontotemporal dementia; HC, healthy controls.

Group comparisons revealed distinct patterns of CSVD marker burden across diagnostic categories (Figure 2). WMH volume differed significantly overall (*p* < 0.001), with both AD and FTD showing a markedly higher burden than HC (both *p* < 0.001), while AD and FTD did not differ significantly from each other (*p* = 0.268). The presence of lacunes also showed a significant global effect (*p* < 0.001), driven by a higher prevalence in AD compared to HC (*p* < 0.001). No significant differences were observed between AD and FTD (*p* = 0.072) or between HC and FTD (*p* = 0.425), suggesting that AD primarily accounts for the overall group effect. A similar pattern was observed for microbleeds, which also differed significantly across diagnostic groups (*p* < 0.001). AD again showed a higher prevalence relative to HC (*p* < 0.001) and FTD (*p* = 0.006), whereas HC and FTD did not differ significantly (*p* = 0.449).

Overall, these results indicate that AD consistently exhibits the greatest burden of CSVD markers, with higher levels across all measures, particularly in comparison with HC. In contrast, FTD shows an intermediate profile, more closely resembling AD for WMHs, while aligning with HC for lacunes and microbleeds.

To examine demographic associations with CSVD markers, regression models including age and sex were fitted for each outcome (Tables S2, S3, and S4). Age showed a consistent positive association with WMH and was also associated with the presence of lacunes and microbleeds across models. Sex did not show significant effects in most models; however, in some cases (microbleeds and WMH in the AD group and WMH in the whole sample), men presented slightly higher odds of microbleeds or WMHs compared to women. These findings indicate that age is the main demographic contributor to CSVD burden in this cohort, whereas sex effects were modest and less consistent.

### 3.3 Relationship between CSVD markers

**FIGURE 3:**
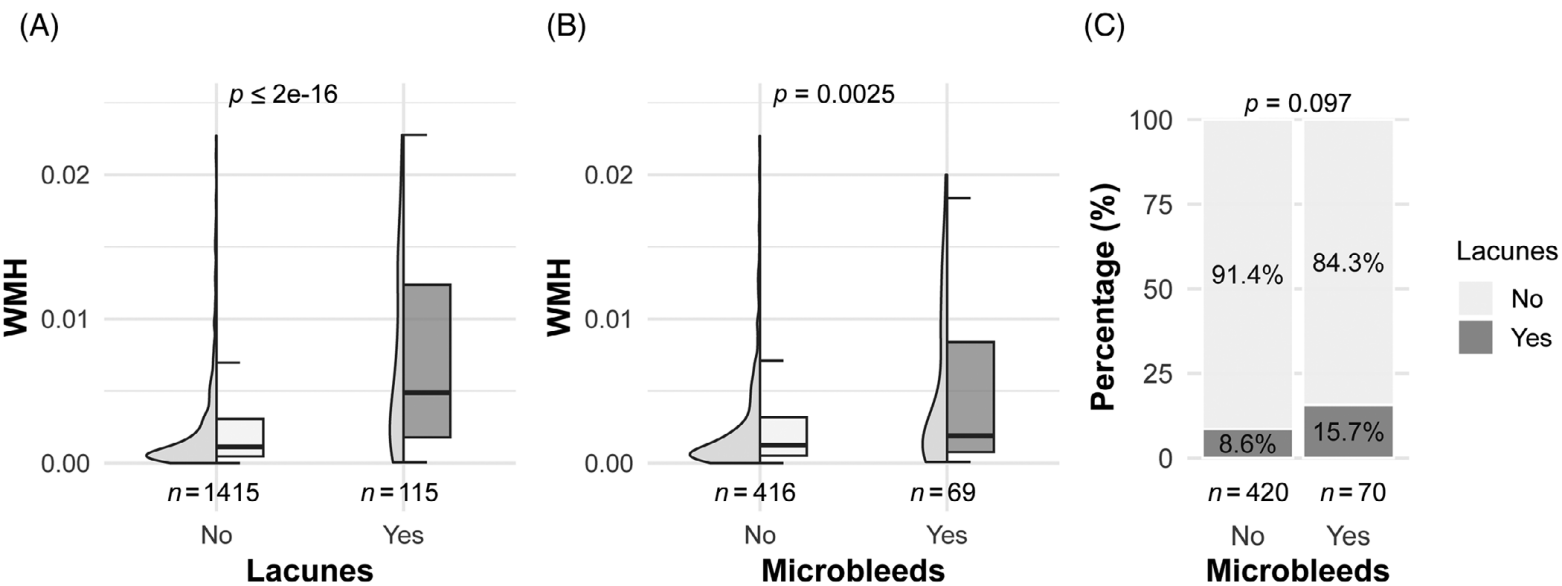
Associations between CSVD markers in full sample. (A) Distribution of WMH burden according to the presence or absence of lacunes. (B) Distribution of WMH burden according to the presence or absence of cerebral microbleeds. (C) Co‐occurrence of cerebral microbleeds and lacunes displayed as percentages. Group comparisons in panels A and B were performed using the Mann–Whitney U test. Associations in panel C were assessed using Pearson's chi‐squared test. WMH, white matter hyperintensity.

We first examined the co‐occurrence of CSVD markers in the whole sample and found that a higher WMH burden was associated with the presence of both lacunes and microbleeds. As shown in Figure 3, participants with lacunes exhibited a substantially higher WMH burden compared to those without lacunes (*p* < 2×10^−16^). A similar but weaker pattern was observed for microbleeds, where WMHs were significantly elevated among individuals with microbleeds, with WMH burden significantly higher among individuals with microbleeds than among those without (*p* = 0.002). Although lacunes and microbleeds co‐occurred in only a minority of participants, lacunes were more prevalent among individuals with microbleeds (15.5%) than among those without microbleeds (8.4%), suggesting partial clustering of CSVD markers; however, this difference did not reach statistical significance (*p* = 0.0916). To complement these whole‐sample analyses, we examined the same associations within each diagnostic group (Figure S1). Briefly, the association between lacunes and higher WMH burden was consistently observed across all diagnoses. In contrast, microbleed–WMH and lacune‐microbleed associations showed a trend only in AD.

Overall, these results demonstrate a robust association between WMH burden and lacunes across the entire sample and within diagnostic groups, whereas associations involving microbleeds were weaker and largely limited to individuals with AD.

### 3.4 Cardiometabolic profiles

Mean systolic BP was 123.1 mmHg (SD = 16.5), with higher values observed in the AD group compared with HC and FTD (HC vs AD *p* < 0.001; HC vs FTD *p*  =  0.9; AD vs FTD *p*  <  0.001), whereas diastolic BP (HC vs AD *p* = 0.6; HC vs FTD *p* = 0.445; AD vs FTD *p* = 0.359) and heart rate (HC vs AD *p* = 1; CN vs FTD *p* = 1; AD vs FTD *p* = 1) were comparable across groups. The prevalence of diabetes was higher in the AD relative to HC group, and no differences were observed between FTD and HC or between AD and FTD (HC vs AD *p* = 0.05; HC vs FTD *p* = 0.13; AD vs FTD *p* = 1). Mean BMI was in the overweight range across all groups, with higher values observed in HC compared with AD and FTD (HC vs AD *p* < 0.001; HC vs FTD *p* <  0.001; AD vs FTD *p* = 0.692). Cumulative smoking exposure, measured in pack‐years, was higher in the AD group compared with HC and also higher in the FTD group compared with HC, while HC showed the lowest smoking exposure (HC vs AD *p* = 0.001; HC vs FTD *p* = 0.006). No significant difference was observed between the AD and FTD groups (*p* = 0.831) (Table 2).

**TABLE 2:** Participants’ cardiometabolic profiles.

Cardiometabolic factor	All (*n* = 1635)	HC (*n* = 773)	AD (*n* = 630)	FTD (*n* = 232)
Systolic BP (mmHg)	123.1 (16.5)	121.2 (16.4)	126.1 (16.7)	120.9 (15.4),
Diastolic BP (mmHg)>	75.0 (9.9)	75.1 (10.0)	75.2 (10.0)	74.0 (9.5)
Heart rate (bpm)>	70.3 (9.7)	70.2 (9.4)	70.3 (10.3)	70.5 (9.2)
Diabetes	14.9%	12.0%	16.7%	16.9%
BMI (kg/m^2^)	26.4 (4.5)	27.4 (4.7)	25.5 (4.0)	25.6 (4.5)
Pack Years (cigarettes/year)	4.6 (12.5)	3.6 (10.7)	5.7 (14.3)	5.0 (11.8)

*Note*: Continuous variables are presented as mean (standard deviation) and categorical variables as percentages. Sample sizes for each group are indicated in parentheses.

Abbreviations: AD, Alzheimer's disease; BMI, body mass index; BP, blood pressure; bpm, beats per minute. FTD, frontotemporal dementia; HC, healthy control.

Overall, AD participants showed a less favorable cardiometabolic profile, characterized by higher systolic BP, greater diabetes prevalence, and increased cumulative smoking exposure, whereas individuals with FTD also showed elevated smoking exposure, and HC exhibited higher BMI values compared with the dementia groups.

### 3.5 Relationship between CSVD indicators and cardiometabolic variables

**FIGURE 4:**
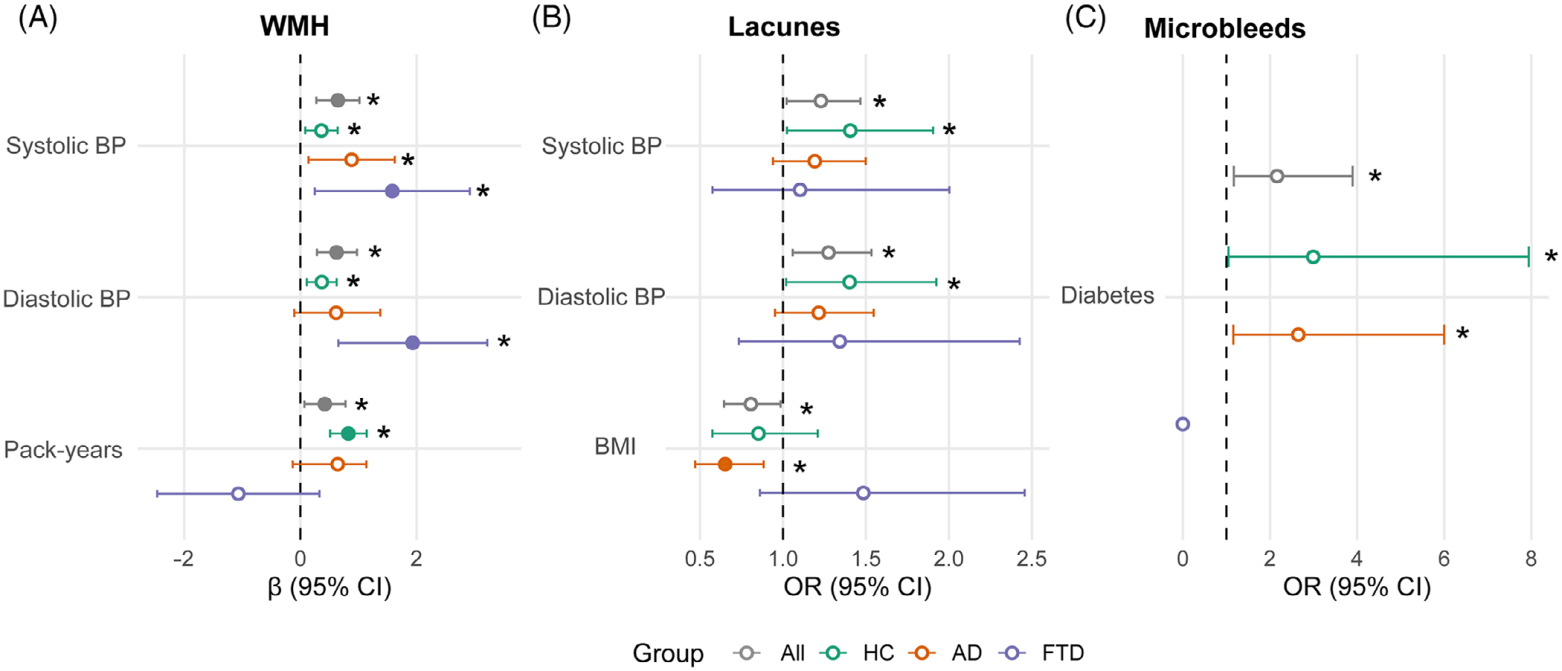
Associations between cardiometabolic risk factors and CSVD markers. Forest plots display associations between cardiometabolic risk factors and (A) WMHs, (B) lacunes, and (C) cerebral microbleeds. Points represent effect estimates (*β* for WMH and odds ratios [ORs] for lacunes and microbleeds), and horizontal lines indicate 95% confidence intervals (CIs). CIs extending beyond the plotting range were truncated for visualization. The dashed vertical line denotes the null effect (*β* = 0 for WMHs; OR = 1 for lacunes and microbleeds). Models were adjusted for age and sex; analyses in the full sample (All) were additionally adjusted for clinical diagnosis, and WMH models were adjusted for total intracranial volume. Colors indicate diagnostic group (All, HC, AD, and FTD). For clarity, only statistically significant associations (*p* < 0.05, uncorrected) are displayed. Complete results are provided in Tables S2, S3, and S4. Filled symbols indicate *p* < 0.05, whereas asterisks denote associations that remained significant after false discovery rate (FDR) correction (*p*FDR < 0.05).

To evaluate the relationship between CSVD markers and classic cardiometabolic risk factors, we conducted regression analyses in the entire sample and separately within each diagnostic group (HC, AD, and FTD). All models were adjusted for age and sex, and volumetric outcomes were additionally adjusted for TIV (Figure 4; all tested cardiometabolic factors are shown in Figure S2).

For WMHs, systolic BP was positively associated with greater WMH burden in the full sample as well as within the AD and FTD groups (all *p*FDR < 0.05). Diastolic BP was also associated with increased WMH volume in the full sample and specifically in FTD (*p*FDR < 0.05), whereas this association did not survive FDR correction in AD. Among HC, cumulative smoking exposure, measured in pack‐years, showed a positive association with WMH burden (*p* < 0.05), which also remained significant in the full sample after FDR correction.

Regarding lacunes, both systolic BP and diastolic BP were associated with their presence in the full sample, but only at the uncorrected level (*p* < 0.05). BMI showed an inverse association with lacunes in AD (*p* < 0.05), but this effect did not survive correction for multiple comparisons.

For cerebral microbleeds, diabetes emerged as a significant predictor in the full sample after FDR correction (*p*FDR < 0.05). This association was also observed within HC and AD at the uncorrected level but did not survive FDR correction in stratified analyses. No other vascular variables were significantly associated with microbleeds.

Sample sizes varied across CSVD due to sequence availability and outcome‐specific exclusions and are reported in Tables S2, S3, and S4.

Overall, these results indicate that BP‐related measures show the most robust associations with WMH burden, smoking exposure is selectively related to WMH in the full sample and in HC, and diabetes is specifically associated with cerebral microbleeds.

### 3.6 Cognitive performance prediction through CSVD markers

We examined the associations between CSVD markers and global cognitive performance (MMSE) in the full sample and stratified by diagnostic group, adjusting for age, sex, education, and clinical diagnosis where appropriate (Table 3).

**TABLE 3:** Association between CSVD markers and global cognitive performance (MMSE).

Group	WMH		Group	Lacunes		Group	Microbleeds	
	*β* (std)	*p* (FDR)		*β* (std)	*p* (FDR)		*β* (std)	*p* (FDR)
All (*n* = 1576)	−0.502 (0.109)	4 × 10^−06^	All (*n* = 1529)	0.105 (0.394)	0.789	All (*n* = 485)	−0.090 (0.425)	0.353
HC (*n* = 753)	0.069 (0.090)	0.806	HC (*n* = 717)	0.086 (0.426)	0.841	HC (*n* = 207)	0.312 (0.312)	0.347
AD (*n* = 604)	−0.844 (0.190)	1.04 × 10^−05^	AD (*n* = 601)	0.099 (0.595)	0.927	AD (*n* = 241)	−0.807 (0.649)	0.429
FTD (*n* = 219)	−0.627 (0.413)	0.261	FTD (*n* = 211)	−0.575 (1.774)	0.746	FTD (*n* = 37)	3.807 (3.162)	0.832

*Note*: Values represent standardized regression coefficients (*β*) with their corresponding standard errors (std), derived from linear regression models. The outcome was global cognition assessed with the Mini‐Mental State Examination (MMSE). Analyses were conducted in the full sample (All) and stratified by diagnostic group (HC, AD, and FTD). All models were adjusted for age, sex, and years of education; models in the full sample were additionally adjusted for clinical diagnosis. *P* values were corrected for multiple comparisons using FDR within each outcome and predictor. *N* indicates the number of participants included in each model.

Greater WMH burden was associated with lower MMSE scores in the full sample and specifically in individuals with AD. In contrast, neither lacunes nor cerebral microbleeds showed significant associations with global cognition in the full sample or within any diagnostic group.

Across domain‐specific cognitive measures, including executive function, episodic memory, language, and processing speed/attention, WMH burden was consistently associated with poorer performance. These associations were observed in the full sample and in AD subjects, where they were most pronounced, whereas more limited associations were detected in HCs (Table S5).

Associations involving lacunes or cerebral microbleeds were sparse and did not survive correction for multiple comparisons.

Overall, these findings underscore WMH burden as the CSVD marker most robustly linked to both global and domain‐specific cognitive impairment, particularly in AD, whereas lacunes and cerebral microbleeds appear to play a more limited and inconsistent role.

## 4 DISCUSSION

To our knowledge, this study provides the first large‐scale neuroimaging characterization of CSVD in a Latin American cohort, integrating MRI markers and cardiometabolic risk factors across healthy older adults, AD, and FTD patients. This contribution is particularly relevant given the region's high prevalence of cardiovascular and metabolic risk factors,^43^, ^44^ rapid population aging, and marked social heterogeneity, alongside its underrepresentation in CSVD research. Most current evidence on CSVD derives from the Global North, leaving open the question of whether established vascular–brain relationships generalize to populations with different demographic, socioeconomic, and health profiles.^43^, ^44^ This concern is reinforced by recent cardiovascular research in Latin America showing that widely used global risk scores systematically misestimate cardiometabolic risk in the region and require local recalibration, despite relying on the same canonical risk factor.^45^, ^46^ Our findings suggest that a similar principle may apply to the brain, where vascular–brain relationships, as indexed by CSVD markers, appear to generalize only partially across populations. In this context, our findings reveal both convergence with and divergence from canonical CSVD patterns.

Several CSVD patterns observed in this cohort align with those reported in large population‐based studies, indicating that the demographic distribution of CSVD markers is broadly comparable across regions. In the Rotterdam Study, most individuals over 60 show some degree of WMHs, while lacunes are present in approximately 7% to 8% of participants and increase sharply with age.^47^, ^48^ In the Framingham Study, lacunes have been reported in ∼10% to 12% of community‐dwelling adults and are strongly associated with age,^49^, ^50^ and microbleeds are observed in approximately 8% to 15% of older adults.^51^, ^52^ Across these large studies, including UK Biobank,^53^ hypertension consistently emerges as the dominant modifiable risk factor, and WMHs are conceptualized as a central marker of overall small vessel burden, correlating with other lesion types within a shared vascular framework. Consistent with this literature and prior Global South reports, such as the BrainLat cohort,^25^, ^46^ age emerged as the strongest correlate of WMH burden, lacunes, and microbleeds in our cohort, underscoring its central role in CSVD accumulation. However, unlike the relatively cohesive CSVD profiles described in population‐based studies, our data revealed a more lesion‐ and diagnosis‐specific organization: WMHs were elevated in both AD and FTD, whereas lacunes and microbleeds more clearly distinguished AD from controls and did not consistently cluster with WMH in FTD. These findings suggest that while the major epidemiological determinants of CSVD are shared across populations, the internal configuration of lesion types may vary across dementia syndromes and clinical contexts. This pattern does not imply distinct underlying mechanisms per se but rather highlights that CSVD phenotypes are not interchangeable across dementia syndromes.

When demographic‐ and diagnostic‐level CSVD patterns are examined alongside cardiometabolic risk factors, associations proved heterogeneous and lesion‐specific rather than uniform. Higher systolic BP predicted greater WMH volume in the full sample and within both dementia groups, while diastolic BP was associated with WMH, particularly in FTD, consistent with evidence linking elevated BP to diffuse WM injury through chronic hypertension‐related arteriopathy.^54^ Smoking exposure was positively associated with WMHs in HCs and the full sample, consistent with the known endothelial and inflammatory consequences of long‐term smoking.^55^ Diabetes predicted microbleeds in the full cohort, as well as in HC and AD groups, supporting prior evidence that glucose dysregulation increased microvascular fragility.^56^ Within AD specifically, BMI showed an inverse association with lacunes, possibly echoing the “obesity paradox” reported in older clinical populations^57^ or the limited sensitivity of BMI relative to measures of central/visceral adiposity.^58^

Cognitive findings supported the functional relevance of this heterogeneity. WMH burden was consistently associated with global cognition and with domain‐specific measures of language, attention, processing speed, and memory. In contrast, lacunes and microbleeds showed weaker and more diffuse associations across domains. This is consistent with prior evidence indicating that the cognitive impact of lacunes depends critically on their number and strategic location rather than their mere presence.^1^, ^2^, ^3^, ^4^, ^5^ Given the exclusion of individuals with large infarcts and overt cerebrovascular syndromes, our cohort likely reflected a relatively moderate vascular burden, in which lacunes were more often clinically silent and less likely to involve cognitively strategic regions.

Although cardiometabolic risk factors are highly prevalent in Latin America,^59^ their associations in this cohort were most consistently captured by WMHs, supporting the view that WMHs reflect the cumulative impact of vascular and metabolic burden across the lifespan.^60^, ^61^ In regions characterized by pronounced social heterogeneity,^62^ lifelong differences in education,^63^ socioeconomic conditions,^64^ and structural inequality^65^, ^66^ may further shape how such risk accumulates over time. In this context, WMHs may emerge as a particularly sensitive marker of overall small vessel burden in heterogeneous populations.

Some limitations should be considered. First, the relatively low prevalence of cerebral microbleeds in some diagnostic subgroups may have limited statistical power to detect more subtle associations, increasing the likelihood of type II error. Second, perivascular spaces, an increasingly recognized component of the CSVD spectrum, were not included in the present analyses and may further refine phenotypic characterization in future work. Third, cardiometabolic risk factors were assessed primarily through clinical history and available measures rather than standardized biomarkers, which may have introduced measurement imprecision and limited detection of subclinical dysfunctions. Importantly, the absence of fasting blood‐based measures limits the ability to detect subclinical or undiagnosed metabolic dysfunction, which is highly prevalent in aging populations and has been consistently associated with structural brain alterations and cognitive decline.^14^, ^36^, ^42^ Finally, the multinational design enhances representativeness but may introduce site‐related variability due to differences in recruitment strategies, diagnostic procedures, and MRI acquisition protocols. Although site effects were not explicitly modeled as hierarchical or random factors in the present analyses, future studies should formally examine potential site‐ and country‐level influences on vascular–brain associations.

## 5 CONCLUSION

This study provides the first large‐scale neuroimaging characterization of CSVD in a Latin American cohort, integrating MRI markers and cardiometabolic risk factors across healthy aging, AD, and FTD. Core associations with age, hypertension, and diabetes were consistent with findings from major international cohorts. However, CSVD organization in this population showed greater lesion‐ and diagnosis‐specific heterogeneity than typically reported, suggesting that it cannot be fully captured by a single, uniform vascular model. These findings underscore the importance of considering diagnostic and population context when interpreting CSVD biomarkers and support expanding vascular research to underrepresented regions.

## CONFLICT OF INTEREST STATEMENT

Authors Florencia Altschuler, Ana Maria Castro‐Laguardia, Verónica Canziani, Adolfo M. García, Martín A. Bruno, Nahuel Magrath, Daniel Franco O'Byrne, Joaquín Migeot, Jessica Hazelton, Marcelo Maito, Andrea Slachevsky, C.D.A., Ignacio Brusco, Nilton Custodio, José Alberto Ávila‐Funes, Diana Matallana, David Aguillón, Hernando Santamaria‐García, Elisa Resende, Leonel Takada, Luis Ignacio Brusco, Jennifer S. Yokoyama, Bruce Miller, Agustín Ibáñez, Vicente Medel, and Cecilia Gonzalez Campo declare no conflicts of interest. Author disclosures are available in the Supporting Information.

## CONSENT STATEMENT

Written informed consent was obtained from all participants or their legally authorized representatives in accordance with institutional guidelines. The study protocol was approved by the ethics committees of all participating institutions and conducted in accordance with the Declaration of Helsinki.

## Supporting information



Supporting information: alz71468‐sup‐0001‐SuppMat.docx

Supporting information: alz71468‐sup‐0002‐ICMJE.pdf
