# Supplementary material for: Mapping the neurovascular landscape in aging and dementia: cerebral small vessel disease markers in a multicenter Latin American cohort
Source: Alzheimers Dement. 2026 May 26;22(5):e71468. doi: 10.1002/alz.71468 (PMC13239890; doi:10.1002/alz.71468)
Supplement: Supplementary file 1 — Supporting information: alz71468‐sup‐0001‐SuppMat.docx [file ALZ-22-e71468-s002.docx]

# Supplementary material and methods

**Structural brain measures**

Structural brain measures revealed clear differences among groups. Total intracranial volume (TIV) was significantly smaller in the AD group compared with both HC and FTD participants (p = 0.002 and p < 0.001, respectively), and HC also showed slightly larger TIV than those with AD (p = 0.010). Consistent with widespread atrophy, both gray matter (GM) and white matter (WM) volumes varied significantly across groups. GM volume was highest in HC, followed by FTD, and lowest in AD (HC > FTD > AD; AD vs. HC p < 0.001, AD vs. FTD p = 0.010, HC vs. FTD p < 0.001). WM volume showed a similar pattern (p < 0.001), with HC exhibiting the largest WM volume, followed by FTD and then AD (all pairwise p < 0.001). In contrast, cerebrospinal fluid (CSF) volume was greatest in FTD, intermediate in AD, and lowest in HC (FTD > AD > HC; global p < 0.001), with significant pairwise differences for AD vs. HC (p < 0.001), AD vs. FTD (p = 0.009), and HC vs. FTD (p < 0.001).

### **Standardization of Cognitive Domains**

Cognitive performance was assessed using a comprehensive neuropsychological battery. To facilitate comparison across cognitive domains and harmonization with previous work, individual test scores were summarized into four cognitive domain composites: language, processing speed/attention, executive function, and episodic memory. The language domain included Animal Fluency, Vegetable Fluency, and the Multilingual Naming Test (MINT). Processing speed/attention included the Trail Making Test Part A (TMT-A) and the Digit Span Forward. Executive function included the Trail Making Test Part B (TMT-B), Digit Span Backward, and phonemic fluency (letters “P” and “M”). Episodic memory included the Craft 21 Word List delayed recall, the Benson figure delayed recall, and the Short-Term Memory Battery (STMB). For the STMB, a composite score was computed as the mean of the three task scores, requiring at least two valid tasks when applicable. Individual test scores were standardized (z-scored) using the HC group as the reference within the cohort. For timed measures (TMT-A and TMT-B), scores were reverse-coded prior to standardization so that higher values indicated better performance. Standardized test scores were then averaged within each cognitive domain to derive composite scores, with higher values reflecting better cognitive performance. Composite scores were computed as the mean of available standardized test scores within each domain.


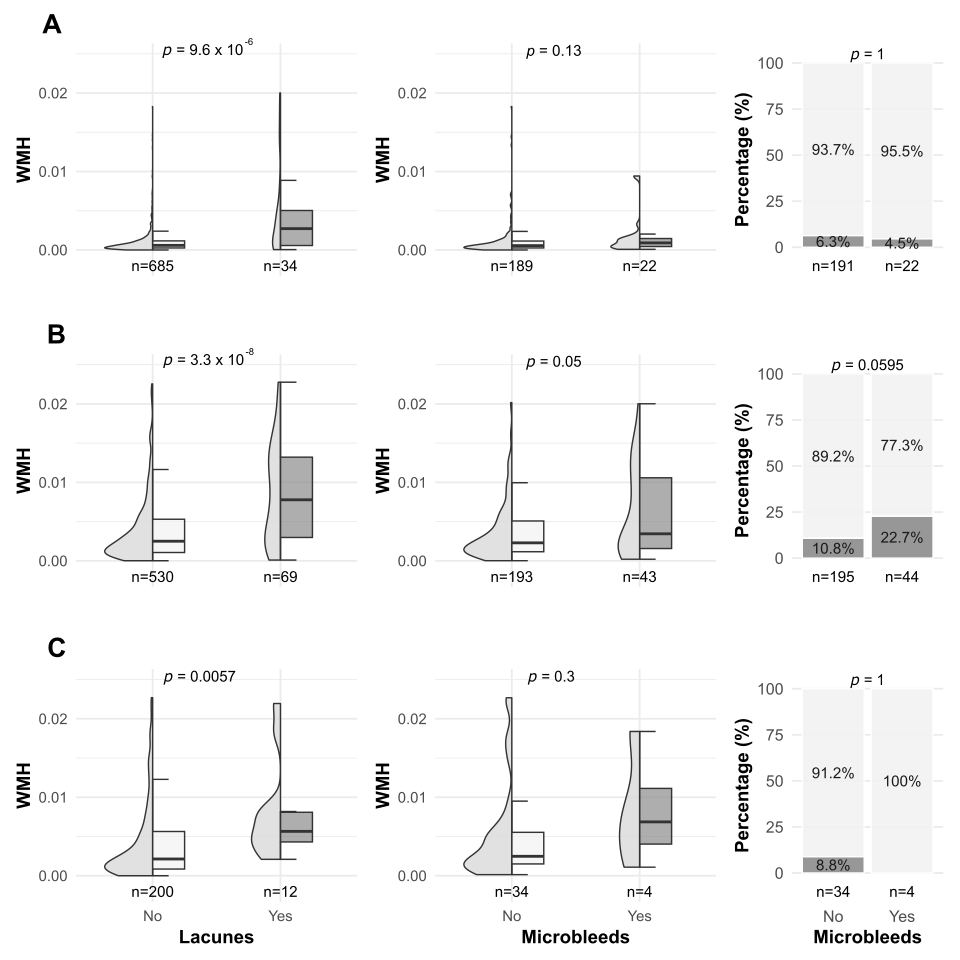


**Figure S1. Associations between cerebral small vessel disease (CSVD) markers across diagnostic groups.**

Panels show the distribution of WMH according to the presence or absence of lacunes (left column) and cerebral microbleeds (middle column), as well as the co-occurrence of lacunes and microbleeds expressed as percentages (right column). Panel A corresponds to HC, Panel B to AD, and Panel C to FTD. Group comparisons of WMH burden were performed using the Mann–Whitney U test, while associations between lacunes and microbleeds were assessed using Pearson’s chi-square test. Sample sizes and p-values are indicated in each panel. WMH = white matter hyperintensities.


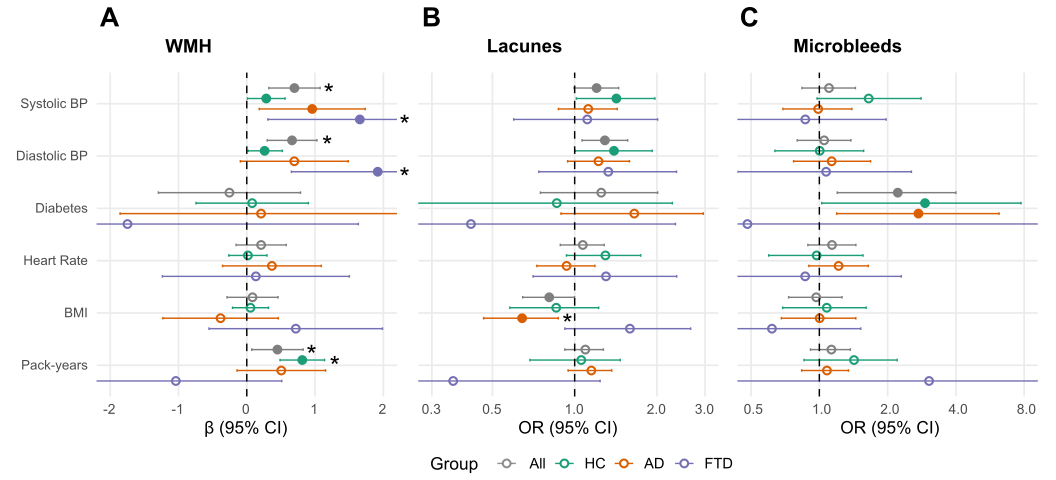


**Figure S2. Associations between cardiometabolic risk factors and CSVD markers.** Forest plots display associations between cardiometabolic risk factors and (A) WMH, (B) lacunes, and (C) cerebral microbleeds. Points represent effect estimates (β for WMH and odds ratios for lacunes and microbleeds), and horizontal lines indicate 95% confidence intervals. Confidence intervals extending beyond the plotting range were truncated for visualization.The dashed vertical line denotes the null effect (β = 0 for WMH; OR = 1 for lacunes and microbleeds). Models were adjusted for age and sex; analyses in the full sample (All) were additionally adjusted for clinical diagnosis, and WMH models were adjusted for TIV. Colors indicate diagnostic group (All, HCN, AD, and FTD). For clarity, only   statistically significant associations (p < 0.05, uncorrected) are displayed. Complete results are provided in Supplementary Tables 2, 3, and 4. Filled symbols indicate p < 0.05, whereas asterisks denote associations that remained significant after false discovery rate (FDR) correction (pFDR < 0.05).

**Table S1.** MRI acquisition parameters by site. The table summarizes scanner manufacturer and model, magnetic field strength (Tesla), image type, sample size (*n*), and voxel dimensions (x, y, z; in mm) for T1-weighted images acquired across participating sites in Argentina, Brazil, Chile, Colombia, Mexico, and Peru. When multiple voxel sizes are reported for a given site, they reflect differences in acquisition protocols across scanners or study phases within the same center.

| **Site** | **Scan** | **Tesla** | **Image type** | **n** | **Voxel size** | | |
| --- | --- | --- | --- | --- | --- | --- | --- |
|  |  |  |  |  | **x** | **y** | **z** |
| Argentina 1 | Philips | 3T | T1 | 3 | 1 | 1 | 1 |
| Argentina 2 | GE Sigma Explorer | 1.5 T | T1 | 146 | 0,9375 | 0,9375 | 1 |
| Brazil 1 | Philips Achieva | 3 T | T1 | 157 | 1 | 1 | 1 |
| Brazil 2 | Siemens Verio | 3 T | T1 | 50 | 1 | 1 | 1 |
|  |  |  |  |  | 1 | 0,5 | 0,5 |
| Chile 1 | Philips Ingenia | 3 T | T1 | 89 | 0,5 | 0,444 | 0,444 |
|  |  |  |  |  | 1 | 1 | 1 |
| Chile 2 | Philips Ingenia | 3 T | T1 | 166 | 1 | 1 | 1 |
|  |  |  |  |  | 0,5 | 0,444 | 0,444 |
| Colombia 1 | Philips Achieva | 3 T | T1 | 171 | 1 | 0,977 | 0,977 |
| Colombia 2 | Phillips Ingenia Elition X | 3 T | T1 | 173 | 0,9 | 0,9375 | 0,9375 |
| Mexico | Philips Achieva | 1.5 T | T1 | 215 | 1 | 1 | 1 |
| Peru | Siemens Skyra | 3 T | T1 | 477 | 1 | 0,977 | 0,977 |

**Table S2. Associations between vascular risk factors and WMH**

Associations between vascular risk factors and WMH burden in the full sample (All) and stratified by diagnostic group (HC, AD, FTD). Values correspond to standardized regression coefficients (β) with 95% confidence intervals derived from linear regression models. All models were adjusted for age, sex and TIV; analyses in the full sample were additionally adjusted for clinical diagnosis. Age and sex estimates correspond to covariates included in the base models and are reported for reference. P values shown correspond to FDR–corrected values. Statistically significant associations after FDR correction are indicated in bold.

| **Group** | **All** | | **HC** | | **AD** | | **FTD** | |
| --- | --- | --- | --- | --- | --- | --- | --- | --- |
|  | **β (95% CI)** | ***p_FDR_*** | **β (95% CI)** | ***p_FDR_*** | **β (95% CI)** | ***p_FDR_*** | **β (95% CI)** | ***p_FDR_*** |
| **Age** | 1.81  (1.404, 2.217) | 1,83x10^-17^ | 1.192 (0.921, 1.463) | 9,45x10^-17^ | 1.99 (1.223, 2.756) | 1,36x10^-6^ | 2.439 (1.157, 3.72) | 0,000675 |
| **Sex (ref:F)** | 1.244  (0.411, 2.078) | 0,0034 | 0.356 (-0.271, 0.982) | 0,265 | 2.01 (0.232, 3.787) | 0,026 | 0.322 (-2.559, 3.204) | 0,826 |
| **Systolic BP (mmHg)** | 0.698  (0.323, 1.074) | 0,001 | 0.285  (0.01, 0.561) | 0,099 | 0.958  (0.182, 1.734) | 0,094 | 1.656  (0.309, 3.003) | 0,049 |
| **Diastolic BP (mmHg)** | 0.663  (0.299, 1.028) | 0,001 | 0.261  (0.001, 0.521) | 0,099 | 0.696  (-0.094, 1.486) | 0,252 | 1.918  (0.654, 3.181) | 0,019 |
| **Heart Rate (bpm)** | 0.21  (-0.157, 0.578) | 0,393 | 0.017  (-0.263, 0.296) | 0,906 | 0.368  (-0.356, 1.092) | 0,448 | 0.133  (-1.236, 1.502) | 0,848 |
| **Diabetes** | -0.254  (-1.295, 0.787) | 0,658 | 0.079  (-0.744, 0.903) | 0,906 | 0.209  (-1.856, 2.274) | 0,842 | -1.747  (-5.128, 1.634) | 0,372 |
| **BMI (kg/m²)** | 0.084  (-0.289, 0.458) | 0,658 | 0.054  (-0.209, 0.318) | 0,906 | -0.384  (-1.229, 0.462) | 0,448 | 0.718  (-0.553, 1.988) | 0,372 |
| **Pack Years (cigarettes/yr)** | 0.449  (0.072, 0.826) | 0,039 | 0.813  (0.487, 1.139) | 0,000 | 0.505  (-0.143, 1.154) | 0,252 | -1.039  (-2.59, 0.512) | 0,372 |

**Table S3. Associations between vascular risk factors and lacunes.**

Associations between vascular risk factors and the presence of lacunes in the full sample (All) and across diagnostic groups (HC, AD, and FTD). Values represent odds ratios (ORs) with 95% confidence intervals estimated using logistic regression models. All models were adjusted for age and sex, and analyses in the full sample were additionally adjusted for clinical diagnosis. Age and sex estimates correspond to covariates included in the base models and are reported for reference. P values shown correspond to FDR–corrected values. Statistically significant associations after FDR correction are indicated in bold.

| **Group** | **All** | | **HC** | | **AD** | | **FTD** | |
| --- | --- | --- | --- | --- | --- | --- | --- | --- |
|  | **β (95% CI)** | ***p_FDR_*** | **β (95% CI)** | ***p_FDR_*** | **β (95% CI)** | ***p_FDR_*** | **β (95% CI)** | ***p_FDR_*** |
| **Age** | 1.979 (1.518, 2.623) | 1,9x10^-6^ | 1.973 (1.338, 3.007) | 0,00195 | 1.574 (1.164, 2.205) | 0,0107 | 2.55 (1.29, 5.626) | 0,0236 |
| **Sex (ref:F)** | 1.132 (0.748, 1.693) | 0,552 | 1.299 (0.593, 2.688) | 0,493 | 1.041 (0.604, 1.755) | 0,881 | 1.209 (0.364, 4.29) | 0,758 |
| **Systolic BP (mmHg)** | 1.203  (0.995, 1.449) | 0,108 | 1.421  (1.014, 1.967) | 0,134 | 1.121  (0.872, 1.431) | 0,438 | 1.112  (0.598, 2.013) | 0,729 |
| **Diastolic BP (mmHg)** | 1.29  (1.064, 1.563) | 0,056 | 1.393  (1.005, 1.922) | 0,134 | 1.222  (0.942, 1.587) | 0,197 | 1.328  (0.739, 2.36) | 0,499 |
| **Heart Rate (bpm)** | 1.071  (0.886, 1.283) | 0,468 | 1.296  (0.933, 1.745) | 0,197 | 0.934  (0.727, 1.186) | 0,585 | 1.302  (0.704, 2.363) | 0,499 |
| **Diabetes** | 1.251  (0.749, 2.013) | 0,448 | 0.86  (0.248, 2.279) | 0,785 | 1.653  (0.891, 2.958) | 0,197 | 0.417  (0.022, 2.34) | 0,499 |
| **BMI (kg/m²)** | 0.807  (0.645, 0.999) | 0,108 | 0.856  (0.579, 1.224) | 0,620 | 0.642  (0.464, 0.871) | 0,035 | 1.593  (0.921, 2.659) | 0,469 |
| **Pack Years (cigarettes/yr)** | 1.094  (0.92, 1.274) | 0,408 | 1.057  (0.686, 1.468) | 0,785 | 1.151  (0.946, 1.367) | 0,197 | 0.359  (0.018, 1.239) | 0,499 |

**Table S4. Associations between vascular risk factors and microbleed.**

Associations between vascular risk factors and the presence of microbleeds in the full sample (All) and across diagnostic groups (HC, AD, and FTD). Values represent odds ratios (ORs) with 95% confidence intervals estimated using logistic regression models. All models were adjusted for age and sex, and analyses in the full sample were additionally adjusted for clinical diagnosis. Age and sex estimates correspond to covariates included in the base models and are reported for reference. P values shown correspond to FDR–corrected values. Statistically significant associations after FDR correction are indicated in bold.

| **Group** | **All** | | **HC** | | **AD** | | **FTD** | |
| --- | --- | --- | --- | --- | --- | --- | --- | --- |
|  | **β (95% CI)** | ***p_FDR_*** | **β (95% CI)** | ***p_FDR_*** | **β (95% CI)** | ***p_FDR_*** | **β (95% CI)** | ***p_FDR_*** |
| **Age** | 1.453 (1.069, 2.014) | 0,0408 | 1.266 (0.823, 1.995) | 0,548 | 1.444 (0.997, 2.197) | 0,0665 | 3.008 (0.727, 22.282) | 0,378 |
| **Sex (ref:F)** | 1.462 (0.85, 2.478) | 0,163 | 0.724 (0.228, 1.946) | 0,548 | 2.237 (1.136, 4.395) | 0,0382 | 0.7 (0.031, 7.005) | 0,775 |
| **Systolic BP (mmHg)** | 1.103  (0.838, 1.442) | 0,717 | 1.65  (0.977, 2.808) | 0,182 | 0.988  (0.691, 1.391) | 0,986 | 0.867  (0.335, 1.969) | 0,995 |
| **Diastolic BP (mmHg)** | 1.05  (0.799, 1.379) | 0,818 | 1.004  (0.637, 1.568) | 0,985 | 1.133  (0.77, 1.682) | 0,795 | 1.071  (0.436, 2.546) | 0,995 |
| **Heart Rate (bpm)** | 1.137  (0.89, 1.449) | 0,598 | 0.973  (0.598, 1.558) | 0,985 | 1.216  (0.899, 1.643) | 0,600 | 0.866  (0.241, 2.297) | 0,995 |
| **Diabetes** | 2.217  (1.199, 4) | 0,056 | 2.923  (1.025, 7.746) | 0,182 | 2.734  (1.192, 6.199) | 0,096 | 0,995 | 0,995 |
| **BMI (kg/m²)** | 0.969  (0.732, 1.259) | 0,818 | 1.078  (0.689, 1.608) | 0,985 | 1.003  (0.68, 1.448) | 0,986 | 0.618  (0.169, 1.523) | 0,995 |
| **Pack Years (cigarettes/yr)** | 1.13  (0.914, 1.369) | 0,598 | 1.422  (0.856, 2.204) | 0,260 | 1.08  (0.836, 1.344) | 0,795 | 3.046  (0.384, 22.929) | 0,995 |

**Table S5. Associations between CSVD markers and domain-specific cognitive performance.**

Associations between markers of CSVD (WMH, presence of lacunes, and presence of cerebral microbleeds) and composite scores of executive function, episodic memory, language, and processing speed/attention. Analyses were conducted in the full sample (All) and stratified by diagnostic group (CN, AD, FTD). Values correspond to standardized regression coefficients (β) with standard errors in parentheses, obtained from linear regression models. All models were adjusted for age, sex, and years of education; analyses in the full sample were additionally adjusted for clinical diagnosis. The number of participants included in each model (n) is reported. P values were corrected for multiple comparisons using the FDR. Statistically significant associations after FDR correction are highlighted in bold.

| **Cognitive domain** | **Group** | **WMH** | | | **Lacunes** | | | **Microbleeds** | | |
| --- | --- | --- | --- | --- | --- | --- | --- | --- | --- | --- |
|  |  | **β (std)** | ***p_FDR_*** | **n** | **β (std)** | ***p_FDR_*** | **n** | **β (std)** | ***p_FDR_*** | **n** |
| Executive function | All | -0.079 (0.018) | 3.0x10^-05^ | 1552 | -0.054 (0.063) | 0.668 | 1505 | 0.011 (0.082) | 0.892 | 475 |
|  | HC | -0.027 (0.025) | 0.36 | 748 | 0.019 (0.102) | 0.845 | 711 | 0.137 (0.132) | 0.302 | 203 |
|  | AD | -0.130 (0.028) | 6.8x10^-06^ | 592 | -0.099 (0.084) | 0.477 | 589 | -0.125 (0.111) | 0.522 | 235 |
|  | FTD | -0.089 (0.051) | 0.17 | 212 | -0.356 (0.227) | 0.236 | 205 | 0.459 (0.378) | 0.320 | 37 |
| Episodic memory | All | -0.099 (0.020) | 2x10^-06^ | 1553 | -0.0862 (0.071) | 0.226 | 1505 | 0.026 (0.087) | 0.763 | 475 |
|  | HC | -0.080 (0.026) | 0.004 | 749 | 0.0674 (0.108) | 0.535 | 712 | -0.071 (0.106) | 0.503 | 203 |
|  | AD | -0.113 (0.031) | 0.001 | 590 | -0.159 (0.093) | 0.088 | 587 | 0.077 (0.130) | 0.552 | 235 |
|  | FTD | -0.174 (0.064) | 0.014 | 214 | -0.311 (0.289) | 0.527 | 206 | 0.003 (0.456) | 0.994 | 37 |
| Language | All | -0.210 (0.027) | 9.84 x10^-14^ | 1552 | -0.162 (0.098) | 0.185 | 1505 | 0.0005 (0.119) | 0.996 | 475 |
|  | HC | -0.115 (0.027) | 4.8x10^-05^ | 748 | -0.135 (0.143) | 0.342 | 588 | -0.009 (0.124) | 0.973 | 203 |
|  | AD | -0.281 (0.046) | 5.37x10^-09^ | 591 | -0.183 (0.113) | 0.215 | 711 | -0.005 (0.179) | 0.974 | 235 |
|  | FTD | -0.254 (0.105) | 0.033 | 213 | -0.288 (0.472) | 0.988 | 206 | -0.559 (0.815) | 0.710 | 37 |
| Processing speed | All | -0.046 (0.20) | 0.026 | 1552 | 0.0004 (0.073) | 0.995 | 1504 | 0.0705 (0.090) | 0.435 | 474 |
|  | HC | -0.014 (0.027) | 0.897 | 748 | 0.067 (0.111) | 0.549 | 711 | 0.241 (0.107) | 0.026 | 203 |
|  | AD | -0.093 (0.036) | 0.009 | 591 | 0.029 (0.107) | 0.780 | 588 | -0.068 (0.135) | 0.834 | 235 |
|  | FTD | -0.010 (0.057) | 0.856 | 213 | -0.582 (0.245) | 0.031 | 205 | 0.3157 (0.505) | 0.896 | 36 |
